# Substance Abuse and Public Health: A Multilevel Perspective and Multiple Responses

**DOI:** 10.3390/ijerph17072610

**Published:** 2020-04-10

**Authors:** T. Wing Lo, Jerf W. K. Yeung, Cherry H. L. Tam

**Affiliations:** Department of Social and Behavioural Sciences, City University of Hong Kong, Hong Kong; t.wing.lo@cityu.edu.hk (T.W.L.); ss.hltam@cityu.edu.hk (C.H.L.T.)

**Keywords:** substance abuse, public health, drug abuse, drug research

## Abstract

Substance abuse has been a thorny public health concern throughout human history. Manifestly, prevention and treatment are the two main strategies commonly adopted to tackle the problem of substance abuse. They are in fact cross-disciplinary, and they relate to the various domains of heredity, biology, psychology, cognitive science, family, social development and cultural structures. This special issue, “Substance Abuse, Environment and Public Health,” has published empirical studies from different regions and countries globally to enhance the international exchange of latest views and findings on the etiology, processes and influences of substance abuse across different domains, through which a multilevel perspective is considered more helpful for analyzing its complex nature, courses and consequences. This in turn suggests the possible need to employ multiple responses dynamically and integratively in the prevention and treatment of substance abuse.

## 1. Introduction

It is apparent that substance abuse is a cross-disciplinary topic of research and concern [1,2], which involves the need to employ concomitantly various theoretical explications and empirical evidence in collaborative efforts to strive for more optimal solutions to limit its contagiousness, and to curb any direct and indirect harm [3,4]. Substance abuse has been described as a “chronic relapsing disease”, with extremely high relapse rates that range from 56.8% to 81.8% [5,6]. Recently, the United Nations reported that “(i)n 2017, an estimated 271 million people, or 5.5 per cent of the global population aged 15–64, had used substances in the previous year” ([7], p.7). This is 11.5% higher than the estimated number of substance-using people in 2012 [8]. Due to the nature of recurrence and the rising number of substance users globally, a continuing upsurge in human, social, health and economic costs in the form of substance-related violence, criminal acts, health care needs, legal orders, rehabilitative services, reduced labor productivity and judicial expenditure is evident [4,6,9]. Undoubtedly, prevention and treatment are two main intervention approaches that have been commonly adopted to tackle substance abuse [2,10], in which the former focuses primarily on enhancing public awareness of the dangers of substance misuse and addiction, and the latter mainly emphasizes helping substance abusers to attain complete abstinence and avoid relapse. Both prevention and treatment of substance abuse are pertinent to public health, as the two approaches need to employ a multilevel perspective to conceptualize and solve fallout generated from drug trafficking, misuse and harm [11,12]. This points to the need to investigate human hereditary, biological, and psychological needs, cognitive and mental conditions, social development and cultural structures simultaneously and interactively.

For a comprehensive understanding of the nature, processes and impact of substance abuse on human individuals and societies as a whole, an international exchange of the latest scholarly views and empirical research findings is needed. This special issue, “Substance Abuse, Environment and Public Health,” aims to promote international exchange of empirical academic works on substance abuse and its related concerns. It includes 14 empirical research articles and one intervention paper from Bosnia, Croatia, Hong Kong, Italy, mainland China, Norway, Poland, Singapore, South Korea, Spain, Slovenia, Sweden and the United States, and covers the topics of substance misuse and addiction amongst various social groups, different types and forms of illicit and legally approved substances and multiple research methods and designs. Importantly, the scholarly works published in this special issue are expected to present an opportunity to enhance the international exchange of cross-disciplinary research and academic inquiries in the prevention and treatment of substance abuse.

## 2. Substance Abuse and Different Social Groups

When researching substance abuse and its harmful effects, researchers predominantly focus on certain social groups with a higher tendency towards substance taking and misuse, such as adolescents and male adults [13,14,15,16,17,18]. This is valid, as they may encounter various demanding life and social challenges, expectations, interpersonal alienation and biological impulses, all of which are relevant to the triggering of their initiation into drug experimentation as a form of self-medication. Substances may also act as a comforting “soul mate” to help users evade hard realities [19,20]. In this special issue, Zubak et al. [21] examined the effects of scholastic factors—for example, grade point averages, school and other unexcused absences and poor behavior—in relation to illicit drug misuse (IDM) and its initiation among adolescents in Bosnia and Herzegovina. Jee et al. [22] investigated the trajectories of different smoking groups of young South Korean male adults and the implication of the habit in their atherosclerotic cardiovascular disease (ASCVD) in middle age. However, substance takers are never restricted to any specific social groups; they can be found in communities of professionals, social talents, elders and university students. Devcic et al. [23] examined socio-demographics, sports-related factors, factors of hesitation, doping-related factors, consumption of dietary supplements, knowledge of doping and predictors of doping behavior in terms of misusing performance-enhancing substances among high-level competitive swimmers in Slovenia. Wang et al. [24] investigated how gender, residential areas and study majors were related to misconceptions about antibiotic use among Chinese university students, which in turn linked to their antibiotic misuse behavior. Through the use of a community-based participatory research design, Walter et al. [25] inquired how work-related musculoskeletal disorders (MSDs) and injuries among US fishing industry workers affected their use of prescription opioids to treat their pain, which in turn exposed them to increased risk of developing substance disorders. Apparently, different social groups are equally susceptible to the risk of substance abuse and addiction [3,4,12], and this is likely to be affected by their specific personal characteristics and environmental conditions. Hence, there is a need for researchers to discover both common and unique precursors germane to different social groups which lead to their substance using behavior.

## 3. Substance Abuse and Its Types and Forms

Substances that are misused or abused can be categorized into two forms. These include illicit and legally approved substances of various types. The most common illicit types of substances include cannabis, amphetamines, ketamine, methamphetamines, cocaine, ecstasy and heroin [2,6], which are largely banned in most countries. However, marijuana products have recently been legalized and commercialized in some northern American and Western states and regions under the umbrella of “control of reasonable use,” which casts a contemplative doubt over the original intent of reducing cannabis-related criminality and public health problems; hence, more research is needed on this subject [26,27,28]. Tobacco and alcohol are two legally approved types of substances that have been widely used by different social groups across different societies and cultures [6,11,29]. Some legally prescribed drugs, such as cough medications and the antibiotics mentioned above, can also be easily misused and abused by the general public, and these too merit the further attention of researchers [2,30].

In this special issue, Lo et al. [31] explored how far using illicit drugs, smoking cigarettes and drinking alcohol predicted sexual misconduct among Macau youths, while simultaneously adjusting for the effects of susceptibility to peer influence and school attachment/commitment. Assari et al. [32] attempted to assess the impact of subjective and objective socioeconomic status on the cigarette smoking and alcohol use of older African Americans by controlling the effects of pertinent covariates, which included demographic factors (age and gender), living arrangement and family type, health insurance status, chronic medical conditions, self-rated health, sick days, depression and chronic pain. Muller et al. [33] investigated changes in exercise and nicotine use among 1464 Norwegian prison inmates by classifying them into harmful and non-harmful substance use pre-incarceration groups, according to the Drug Use Disorders Identification Test (DUDIT) and the Alcohol Use Disorders Identification Test (AUDIT), both of which are commonly used by healthcare practitioners and researchers to assess the severity of illicit drug and intoxicant use. Wang et al. [34] analyzed the sources of antibiotics leftovers in the home and the risk factors of keeping them in relation to antibiotic self-medication among Chinese university students. Taken together, the relationships between the use of illicit drugs and legally approved substances are complex and intertwined or mutually reinforcing [35,36]. They may be affected by the personal circumstances and environmental conditions of the abusers, and may cause other forms of behavioral maladjustment [17,37,38]. Nevertheless, our current understanding of this complicated phenomenon of substance abuse is limited, and so more cross-disciplinary research is again recommended.

## 4. Researching Substance Abuse: Methods and Designs

As has been mentioned, substance abuse is a public health concern that involves human biological and physical needs, psychosocial demands, cognitive and spiritual fulfillment, and environmental formulations. Therefore, cross-disciplinary research using different methodologies and designs is much needed to scrutinize substance abuse in respect of etiology, maintenance, consequences, abstinence and relapse. Generally speaking, empirical studies using quantitative methods are more common than research involving qualitative inquiry, analysis of secondary data and/or documentary inspection [16,39,40]. In fact, research based on a range of methods and designs is useful in enhancing our comprehension of the nature and impact of substance abuse from different perspectives. This special issue incorporates empirical studies conducted by quantitative, qualitative and documentary methods. For quantitative research designs, study findings based on a representative sample or any of the random sampling procedures are desirable, and can strengthen empirical evidence and provide greater external validity [41]. For example, Oh et al. [42] investigated whether those who had current or previous experience of facial flushing would drink for different primary reasons, compared with those who had no experience of facial flushing. The sample comprised 4590 college students who were recruited by stratified random sampling procedures proportionately in 82 colleges in South Korea. There are other empirical studies in this special issue that similarly used a representative sample [21,24,31,34]. However, using quantitative methods to survey empirically the attitudes and behaviors of certain health and human service professional groups is less likely to require a representative sample, and so it is necessary to use non-probabilistic sampling procedures such as quota, purposive or snowballing sampling designs. Molina-Mula et al. [43] analyzed the attitudes and perceptions of emergency and mental health nurses with regard to alcoholics. Their findings will hopefully help to develop appropriate professional and clinical responses to substance abuse.

Qualitative research methods can help reveal in-depth and formative information related to the processes and development of substance abuse. For their qualitative study, Chan et al. [44] interviewed 67 drug abusers to explore how their psychological experiences—with special emphasis on interpersonal relatedness—affected their drug taking and relapse behaviors. Walter et al. [25] used qualitative interviewing to examine knowledge of and attitudes towards opioid use among 21 fishing industry workers in the US. In addition, use of secondary data or documentary information can efficiently and objectively assist in the transition processes of substance users. For example, Asharani et al. [45] employed and analyzed recorded data from the Registry of Birth and Death, Immigration and Checkpoint Authority of Singapore to investigate the unnatural deaths of 42 treatment seekers of substance addiction between 2011–2015. Their findings provide evidence of the lethal consequences of substance abuse in an unobtrusive manner. Moreover, Chmielowiec et al. [46] examined the relationship between the mesolimbic dopamine system and addiction in a group of 299 addicted subjects and another group of 301 non-addict controls by analyzing two polymorphisms in their genes (a variable number of tandem repeats in DRD4 and DAT1), which are mainly responsible for dopaminergic transmission, representing a human reward system that is closely related to substance abuse and misuse. It is clear that research using different methods and designs is useful in fortifying and enhancing currently established concepts and knowledge of substance abuse. Therefore, more novel research methods and designs should be encouraged, so that patterns of substance abuse can be more efficiently dissected.

## 5. Conclusions

Substance abuse has been an issue of public health for hundreds of years [47]. Nevertheless, professionals and researchers of different domains tend to adopt a one-dimensional view based on their particular expertise when examining, explaining and trying to find solutions to this complex problem [10,48,49]. Thus, various and often competing perspectives rooted in the paradigms of heredity, biology, psychology, cognitive science, family, social development and cultural structures can exist simultaneously, thereby unwittingly compounding the problem [1,2,39,50]. However, as substance abuse is composed of layers of individual development, family and social influences, cultural values and environmental conditions, a multilevel perspective is needed to analyze its etiology, maintenance and consequences. Various theories and models from different scholarly paradigms at different levels of social systems should be employed concomitantly to help examine and resolve the issues as part of a dynamic and comprehensive process [2,3,12]. Employing such a multilevel perspective requires researchers and practitioners to explore the interaction of hereditary, physical, psychological, cognitive, mental, family, social, cultural and environmental factors, and to show exactly how such synergy leads to and/or maintains substance use and addiction. Doing so will help in the design of improved multiple responses to the fallout from substance abuse.

As substance abuse is never limited to particular social groups in human societies, it is essential to understand the unique psychological, personality, cognitive, socioeconomic, familial and cultural differences of various social groups, and to explore what common and unique characteristics they hold in terms of the initiation, processes and consequences of substance abuse [4,6]. If researchers, service practitioners, educators and policy makers were able to understand the common and unique etiological causes and stimulants that incur experimentation and the subsequent maintenance of substance abuse, more effective prevention and treatment strategies and programs could be introduced. Furthermore, because each society or nation is comprised of multiple differing social groups, a knowledge and understanding of their unique cultural and ethnic structures would be empirically useful for researchers trying to unearth the common and distinct etiological causes and stimulants of substance use and abuse. This is a largely unchartered area of research.

The abuse of different types and forms of substances may generate different levels of addiction and harm [2,51], which in turn may trigger distinct social maladjustment and craving behaviors [12,52]. Therefore, future research should discern and clarify the effects of different types and forms of substances on the progress, abstinence and relapse of addicts; this would lead to a better comprehension of the nature and impact of substance abuse. Quantitative methods and designs should be adopted to this end, in addition to other methods and designs that will broaden our perspectives on the topic. In other words, future addiction research should consider the employment of mixed-method designs to investigate the nature of different types and forms of substances and their effects on different social groups. Furthermore, the interaction between the biological, individual, family, social and cultural factors that lead to substance abuse is worthy of research, but will require more advanced methodological designs and mathematical and statistical procedures.

The processes and consequences of substance abuse can be seen to evolve in step with social, technological and cultural developments [4,39]. The patterns and forms of substance abuse can vary according to different social groups. Therefore, comparative and longitudinal research is more useful and insightful in helping to reveal its precarious and dynamic influences. In fact, polysubstance abuse—in which substance addicts expect to achieve higher substance-synergy effects of enjoyability by simultaneously abusing multiple types of drugs and substances—has become more common in the past decade [53,54]. This apparently presents an even greater challenge to treatment and healthcare services. In the face of this new phenomenon, the role of empirical research becomes more pivotal in helping to configure effective approaches and solutions.

In conclusion, substance abuse has long been a thorny public health problem, and it continues to evolve. Multiple responses supported by the employment of a multilevel research perspective are needed. Cross-disciplinary collaboration and concerted research are urgently required if we are to optimize our current strategies and remediation.
